# Spatial alterations of *De Novo* purine biosynthetic enzymes by Akt-independent PDK1 signaling pathways

**DOI:** 10.1371/journal.pone.0195989

**Published:** 2018-04-18

**Authors:** Danielle L. Schmitt, Anand Sundaram, Miji Jeon, Bao Tran Luu, Songon An

**Affiliations:** Department of Chemistry and Biochemistry, University of Maryland Baltimore County (UMBC), Baltimore, Maryland, United States of America; Queen Mary University of London, UNITED KINGDOM

## Abstract

A macromolecular complex of the enzymes involved in human *de novo* purine biosynthesis, the purinosome, has been shown to consist of a core assembly to regulate the metabolic activity of the pathway. However, it remains elusive whether the core assembly itself can be selectively controlled in the cytoplasm without promoting the purinosome. Here, we reveal that pharmacological inhibition of the cytoplasmic activity of 3-phosphoinositide-dependent protein kinase 1 (PDK1) selectively promotes the formation of the core assembly, but not the purinosome, in cancer cells. However, alternative signaling cascades that are associated with the plasma membrane-bound PDK1 activity, including Akt-mediated cascades, regulate neither the core assembly nor the purinosome in our conditions. Along with immunofluorescence microscopy and a knock-down study against PDK1 using small interfering RNAs, we reveal that cytoplasmic PDK1-associated signaling pathways regulate subcellular colocalization of three enzymes that form the core assembly of the purinosome in an Akt-independent manner. Collectively, this study reveals a new mode of compartmentalization of purine biosynthetic enzymes controlled by spatially resolved signaling pathways.

## Introduction

**Fig 1:**
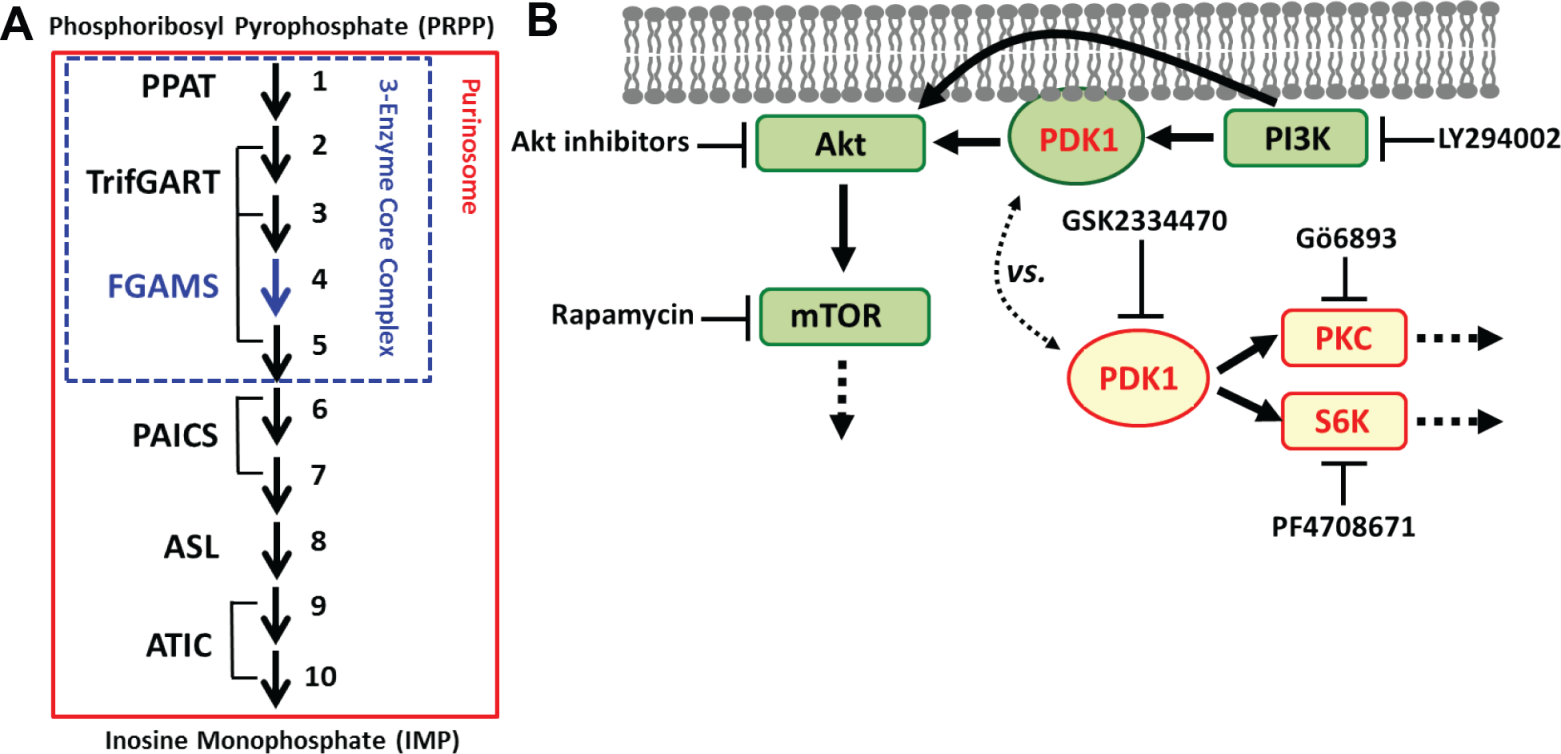
De novo purine biosynthesis and PDK1-associated signaling pathways. (A) Biosynthesis of inosine monophosphate (IMP) from phosphoribosyl pyrophosphate (PRPP) is catalyzed by six enzymes and their assemblies. A 3-enzyme core assembly (blue box) catalyzes the first half of the pathway while the purinosome (red box) regulates the entire pathway. (B) 3-Phosphoinositide-dependent protein kinase 1 (PDK1) is recruited to the plasma membrane upon activation of phosphoinositide 3-kinase (PI3K). Subsequently, PI3K and PDK1 activate Akt for various signaling cascades, including the mammalian target of rapamycin (mTOR). Alternatively, cytoplasmic PDK1 phosphorylates several kinases, including protein kinase C (PKC) and p70 ribosomal protein S6 kinase (S6K), in an Akt-independent manner. Pharmacological inhibitors used in this work are also indicated.

Purines are associated with a variety of cellular processes as secondary messengers, cofactors and nucleic acids [1]. Inosine monophosphate (IMP), a metabolic product of purine biosynthesis, is synthesized in one step by a purine salvage enzyme, hypoxanthine-guanine phosphoribosyltransferase (HPRT), which conjugates hypoxanthine with phosphoribosyl pyrophosphate (PRPP). The salvage pathway appears to be sufficient to meet the cellular demand for purines in normal healthy cells [2]. On the other hand, *de novo* purine biosynthesis also contributes to cellular purine levels by converting PRPP to IMP in ten chemical reactions (Fig 1A). Briefly, six enzymes, three of which are multifunctional enzymes, are responsible for the sequential reactions; i.e. PRPP amidotransferase (PPAT; step 1), a trifunctional enzyme (TrifGART; steps 2, 3 and 5) with glycinamide ribonucleotide synthetase, glycinamide ribonucleotide transformylase and aminoimidazole ribonucleotide synthetase activities, formylglycinamidine ribonucleotide synthase (FGAMS; step 4), a bifunctional enzyme (PAICS; steps 6 and 7) with carboxyaminoimidazole ribonucleotide synthetase and succinylaminoimidazolecarboxamide ribonucleotide synthetase activities, adenylosuccinate lyase (ASL; step 8); and a bifunctional enzyme (ATIC; steps 9 and 10) with aminoimidazolecarboxamide ribonucleotide transformylase and IMP cyclohydrolase activities. Since *de novo* purine biosynthesis is often upregulated in cancer cells, the enzymes in the *de novo* pathway have been targeted for anti-cancer chemotherapy [3,4].

However, it is only recently that a subcellular localization-function relationship for *de novo* purine biosynthesis has been investigated to provide novel insights of how the pathway is spatially and thus functionally regulated at subcellular levels [5,6]. A total of six *de novo* purine biosynthetic enzymes compartmentalize into a macromolecular metabolic complex, the purinosome (Fig 1A), under purine depleted conditions in HeLa cells [7] as well as in Hs578T cells [8]. The purinosome is proposed to consist of a 3-enzyme core assembly that catalyzes the first half of the pathway, steps 1 to 5 by PPAT, TrifGART and FGAMS (Fig 1A) [8,9]. Purinosome formation is also correlated with the increased rate of *de novo* purine biosynthesis [10] as well as the increased pool of its final product, IMP [11]. Accordingly, the purinosome formation represents the upregulation of *de novo* purine biosynthesis in human cells. Meanwhile, we further demonstrated that one of the three core enzymes, FGAMS, can be spatially sequestered in the cytoplasm, resulting in the downregulation of *de novo* purine biosynthesis [12]. Indeed, our understanding of the localization-function relationship for *de novo* purine biosynthesis has been drastically advanced in recent years. However, it has remained elusive whether the 3-enzyme core assembly itself can be selectively controlled in the cytoplasm to possibly diversify the functional contributions of the pathway to cellular metabolism.

Meanwhile, 3-phosphoinositide-dependent protein kinase 1 (PDK1), a constitutively active serine/threonine kinase, has been found in both the cytoplasm and the plasma membrane of human cells (Fig 1B) [13,14]. The C-terminal pleckstrin homology domain of PDK1 enables its translocation from the cytoplasm to the plasma membrane in response to increased levels of phosphatidylinositol-3,4,5-trisphosphate [15]. Subsequently, Akt (or protein kinase B) on the plasma membrane and thus the mammalian target of rapamycin (mTOR) are activated to promote cell growth and proliferation. Alternatively, PDK1 is also involved in a variety of cellular processes through its cytoplasmic substrates, including protein kinase C (PKC), p70 ribosomal S6 kinase (S6K), protein kinase A, and serum glucocorticoid kinase [14,16]. Such Akt-independent PDK1 signaling pathways appear to play an essential role in the anchorage-independent growth of breast cancer cells during migration and invasion [17–20]. Therefore, the PDK1-mediated signaling cascades are distinctively divided into at least two pathways based on its subcellular locations and Akt participation [13,14,18].

Due to the functional roles of PDK1-associated signaling pathways in various cellular processes, particularly including its relationship with *de novo* pyrimidine biosynthesis [21,22], we have been interested in establishing an interaction network between PDK1-associated signaling pathways and *de novo* purine biosynthesis. Using a series of pharmacological inhibitors and time-lapse live-cell imaging approaches, we have found that the inhibition of phosphatidylinositol-3-kinase (PI3K), Akt, or mTOR, whose cascade starts at the plasma membrane, have no impact on the subcellular distribution of *de novo* purine biosynthetic enzymes in human cancer cells. On the other hand, pharmacological inhibitions of PDK1 or PKC and S6K, which are downstream kinases of the cytoplasmic PDK1 activity, promote the formation of the 3-enzyme core assembly for *de novo* purine biosynthesis. Our results from immunocytochemistry using a small mollecule and knock-down studies using small-hairpin interfering RNAs against PDK1 (shRNA_PDK1_) corroborate with our phamacological studies, supporting the formation of the 3-enzyme assembly. Collectively, we demonstrate that the enzymes of *de novo* purine biosynthesis are tightly associated with Akt-independent PDK1 signaling pathways in a subcellular location-specific manner.

## Materials and methods

### Materials

All plasmids expressing *de novo* purine biosynthetic enzymes with enhanced monomeric green fluorescent protein (mEGFP) or monomeric orange fluorescent protein (mOFP) were previously prepared [7,12].

### Chemicals

Various small molecules were purchased for this study and dissolved in dimethyl sulfoxide (DMSO) solution: GSK2334470 (Sigma), Gö6893 (Calbiochem), PF4708671 (Torcis), Akt inhibitor X (Calbiochem), Akt inhibitor VI (H-AVTDHPDRLWAWEKF-OH; Calbiochem), LY294002 (Calbiochem), and rapamycin (Calbiochem). Cellular staining reagents were purchased from Molecular Probes (Fisher Scientific), which included 1,1’-dihexadecyl-3,3,3’,3’-tetramethylindocarbocyanine perchlorate (DiIC_16_(3); Cat# D384), and MitoTrackerOrange CMTMRos (Cat# M7510).

### Mammalian cell culture

Human cervical adenocarcinoma cell line (HeLa) and human breast carcinoma cell line (Hs578T) were obtained from the American Type Culture Collection (ATCC). HeLa and Hs578T cells were maintained in the ATCC-recommended culture conditions: Minimum Essential Medium (MEM; Mediatech, Cat# 10-010-CV) or Dulbecco’s modified eagle medium (DMEM; ATCC, Cat#30–2002) supplemented with 10% fetal bovine serum (FBS) (Atlanta Biological or Sigma Aldrich) and 50 μg/ml gentamycin sulfate (Corning), respectively. Both cell lines were maintained in a HeraCell CO_2_ incubator (37 ^o^C, 5% CO_2_ and 95% humidity).

### Transfection

Cells were plated on 35 mm glass bottomed petri dishes (MatTek) or 8-well chambers (LabTek) without antibiotics. Cells were transiently transfected on the next day using Lipofectamine^®^ 2000 (Invitrogen) to express mEGFP-tagged proteins and/or mOFP-tagged proteins in HeLa or Hs578T cells. After incubation with Lipofectamine-DNA plasmid complexes for ~5 hours, the cells were washed and incubated with fresh growth media in a HeraCell CO_2_ incubator (37 ^o^C, 5% CO_2_ and 95% humidity) for 20–24 hours. On the day of imaging, the transfected cells were washed three times with imaging solution (20mM HEPES (pH 7.4), 135 mM NaCl, 5 mM KCl, 1 mM MgCl_2_, 1.8 mM CaCl_2_ and 5.6 mM glucose), followed by at least one hour incubation at ambient temperature prior to imaging.

### Fluorescence live-cell imaging and image analysis

All samples were imaged at ambient temperature (~25 ^o^C) with a 60x 1.45 NA objective (Nikon CFI PlanApo TIRF) using a Photometrics CoolSnap EZ monochrome CCD camera mounted onto a Nikon Eclipse Ti inverted C2 confocal microscope. Wide field imaging was carried out using the following filter sets from Chroma Technology: detection of mEGFP was done using a set of Z488/10-HC cleanup, HC TIRF Dichroic, and 525/50-HC emission filter. Similarly, mOFP detection was done using a set of Z561/10-HC cleanup, HC TIRF Dichroic, and 600/50-HC emission filter. For confocal imaging, photomultipliers mounted to the microscope were used for image acquisition. For mEGFP detection, we used a JDSU argon ion 488 nm laser line with a 488/561 nm dichroic mirror and 525/50 nm emission filter. Data was collected using NIS-Elements AR 4.13.04 (Nikon). Each small molecule was diluted with DMSO to achieve respective final concentrations of GSK233470 (11–33 μM), Gö6983 (57 nM), PF4708671 (64 μM), Akt inhibitor X (13 μM), Akt inhibitor VI (0.5–27 μM), LY294002 (26 μM) and rapamycin (650 nM). Time-lapse imaging was performed by acquiring images before and after the addition of drugs at various time points. Control experiments were also carried out using DMSO (1–5 μl).

Captured images were analyzed using the *FIJI* software package (National Institutes of Health). The percentage of cells showing the colocalization of two enzymes was determined by dividing the number of cells displaying co-clusters by the total number of dually transfected cells. In addition, Pearson’s correlation coefficient for dually transfected cells was measured using the CoLoc2 module in the *FIJI* software package.

### Knock-down of PDK1

HeLa cells were dually transfected following the above procedure with FGAMS-mOFP and a bicistronic plasmid expressing shRNA targeting PDK1 (shRNA_PDK1(1)_, AAACCCTTGGCACCAGTTTGT; or shRNA_PDK1(2)_, TCGACCAGCGGCCAAGAATTT) or a scrambled sequence (shRNA_scrambled_, GGAATCTCATTCGATCGATAC) under a U6 promoter, and GFP under a CMV promoter, allowing for selection of shRNA-expressing cells (Qiagen Cat# 336311 KH00783G). At least 24 hours later, cells were washed with our imaging buffer and imaged as described above.

### Immunocytochemistry

HeLa cells that were plated on 35 mm glass-bottom petri dishes (MatTek) in an antibiotic-free medium, were washed with the imaging buffer and fixed with 100% methanol at -20 ^o^C for 15 minutes, or treated with GSK2334470 and then fixed. Following fixation, cells were blocked with 5% normal donkey serum for 30 min at room temperature. Cells were then incubated with primary antibodies against TrifGART (Rabbit anti-TrifGART (1:300)) [7] or FGAMS (Rabbit anti-PFAS (1:300, Bethyl Laboratories, Cat#A304-218A)). Subsequently, cells were incubated with Cy3-conjugated secondary antibody (Donkey anti-Rabbit Cy3 (1:500, Jackson Immunoresearch)). All antibody dilutions were prepared in 5% normal donkey serum. Cells were washed and imaged in 1xDPBS (pH 7.4) (Corning # 21-030-CV).

### Western blotting

Lawns of HeLa cells were transfected with shRNA_PDK1_ or shRNA_scrambled_. Harvested cell pellets were lysed in lysis buffer (50 mM sodium phosphate pH 8, 300 mM NaCl, 0.5 mM DTT, 1% Nonidet P40) containing cocktails of protease inhibitors (Pierce Protease Inhibitor Mini Tablets #88666). Cell lysates were then concentrated (10 kDa MWCO Filters, Millipore #UFC501024) and run on 5–8% SDS-PAGE gels. Proteins were transferred to a PVDF membrane using a BioRad Semi Dry Transfer System. The PVDF membrane was blocked using LI-COR PBS Blocking Buffer (LI-COR #927–40100) and incubated overnight with primary antibodies (Rabbit anti-PDK1 (1:1000, Cell Signaling)), followed by a brief incubation with mouse anti-actin antibody (1:10000, Sigma). Subsequently, secondary antibodies conjugated with near-IR dyes were treated (Donkey anti-Rabbit-AlexaFluor790 (1:10,000, Jackson Immunoresearch), and Donkey anti-Mouse-DyLight690 (1:10,000, Cell Signaling)). The membranes were then imaged using a LI-COR Odyssey Sa Near-IR imager. Blots were analyzed using ImageStudioLite (LI-COR).

### Fluorescence recovery after photobleaching (FRAP)

FRAP was performed using the instrumentation outlined above. For photobleaching, the argon ion 488 nm laser was used at 75% power for 1 sec. Before bleaching at least 10 images were obtained, and post-bleaching images were obtained approximately every second for at least 100 sec. Individual fluorescence recovery was normalized and corrected for background photobleaching and fitted as we described previously [8,12].

## Results

We have extensively performed a series of pharmacological studies to map a pathway communication network between *de novo* purine biosynthetic enzymes and PDK1 signaling pathways in HeLa and Hs578T cells. Both cancer cell lines were maintained in nutrient-rich conditions (Materials and Methods), where all the enzymes in *de novo* purine biosynthesis diffusively stain the cytoplasm in the absence of exogenous stimuli [7,8].

### Inhibition of PDK1, PKC and S6K promoted the assembly of FGAMS in HeLa and Hs578T cells

**Fig 2:**
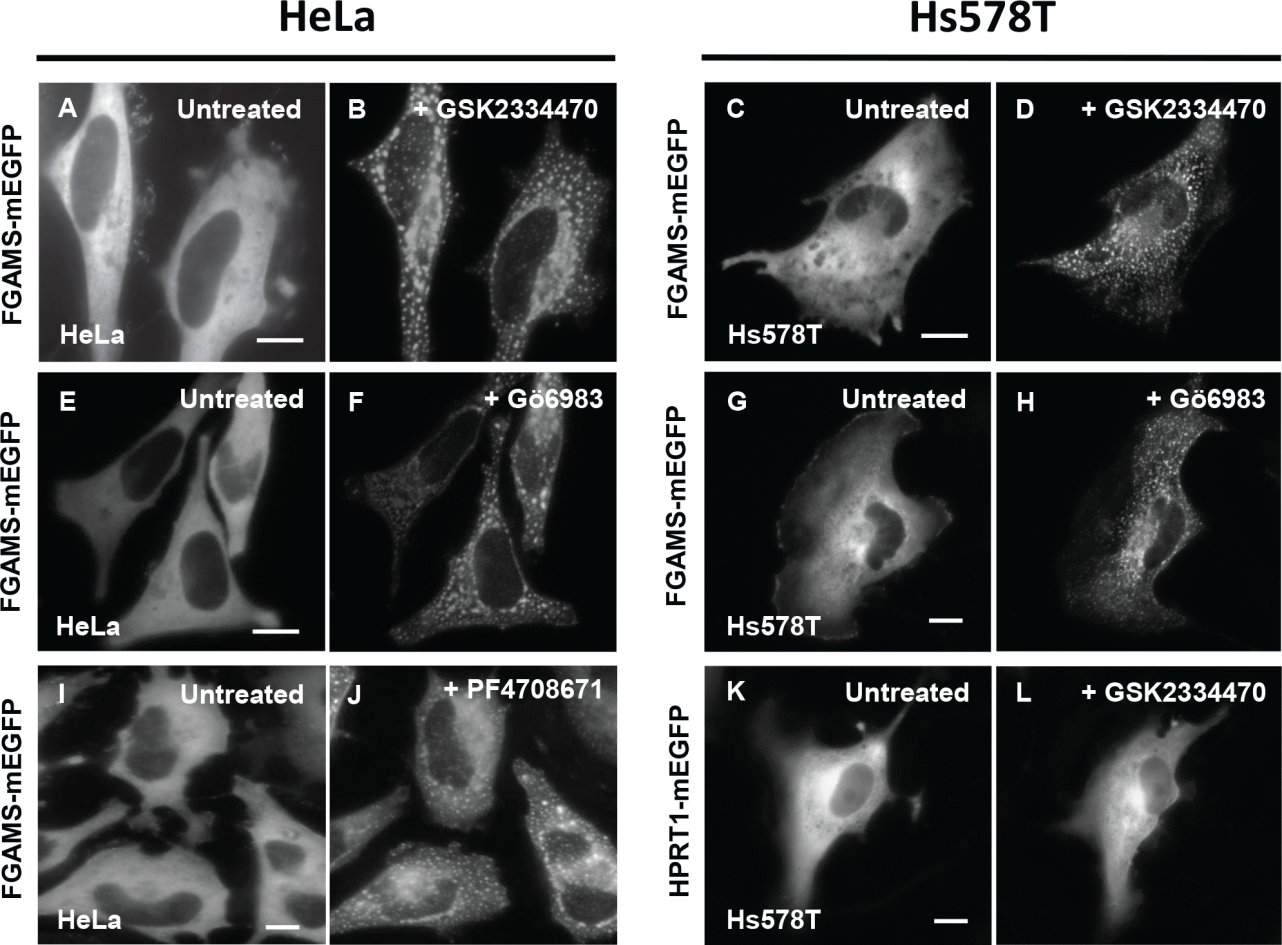
Regulation of cytoplasmic activity of PDK1 with small molecules promotes clustering of FGAMS-mEGFP. HeLa and Hs578T cells expressing FGAMS-mEGFP were treated with a PDK1 inhibitor GSK2334470 (A-D; *N*_HeLa_ = 158 and *N*_Hs578T_ = 791) or a PKC inhibitor Gö6983 (E-H; *N*_HeLa_ = 623 and *N*_Hs578T_ = 146) for 4–5 hours. HeLa cells expressing FGAMS-mEGFP were also treated with a S6K inhibitor PF4708671 (I-J; *N*_HeLa_ = 623). As a negative control, Hs578T cells expressing purine salvage enzyme HPRT1-mEGFP were treated with GSK2334470 for 5–6 hours. Unlike FGAMS-mEGFP, clustering of HPRT1-mEGFP was not observed (K-L). The representative images were selected from at least three independent imaging sessions. *N* indicates the number of the cells we have imaged in our study. Scale bar, 10 μm.

To examine subcellular locations of *de novo* purine biosynthetic enzymes in the presence of phamacological inhibitors, we transfected the plasmid expressing monomeric enhanced green fluorescent protein-tagged FGAMS (FGAMS-mEGFP) in both HeLa and Hs578T cells. We then monitored the subcellular distribution of the enzyme under fluorescence live-cell imaging before and after pharmacological inhibition. First, a specific and potent inhibitor of PDK1, GSK2334470 (11–22 μM) [14], robustly promoted cytoplasmic assemblies of FGAMS in ~90% of HeLa and ~80% of Hs578T cells after the drug addition (Fig 2A–2D). Given that Akt inhibition with A443654 (Abbott) was previously shown to have no effect on FGAMS assembly or disassembly in HeLa cells [23], we postulated that cellular signals promoting the FGAMS assembly in the presence of the PDK1 inhibitor would be transmitted in an Akt-independent manner.

Accordingly, we targeted two downstream kinases of Akt-independent PDK1 activity. When we applied Gö6983 (57 nM), a potent inhibitor of PKC [24,25], cytoplasmic FGAMS assemblies were promoted in ~85% of HeLa and ~70% of Hs578T cells (Fig 2E–2H). Alternatively, when we administered a potent inhibitor of S6K, PF4708671 (64 μM) [26], we were able to monitor the formation of FGAMS assemblies in HeLa cells (Fig 2I and 2J), supporting the importance of the Akt-independent activity of PDK1. As controls, individual addition of GSK2334470 or Gö6983 to HeLa and Hs578T cells expressing mEGFP alone (Panel A-F in S1 Fig) or HPRT1-mEGFP (Fig 2K and 2L) had no impact on their subcellular localizations. Application of vehicle (e.g. DMSO) did not induce clusters of FGAMS-mEGFP in Hs578T cells (Panel G-H in S1 Fig) as well as in HeLa cells as previously reported [12,23]. Please, note that the treatment of the small molecules to HeLa and Hs578T cells (i.e. 4–6 hours) did not substantially influence cell viability, growth or differentiation. Collectively, we conclude that the inhibition of PDK1 or its downstream kinases promote the clustering of FGAMS in HeLa and Hs578T cells in an Akt-independent manner.

### Knock-down of PDK1 induced the assembly of FGAMS in HeLa cells

**Fig 3:**
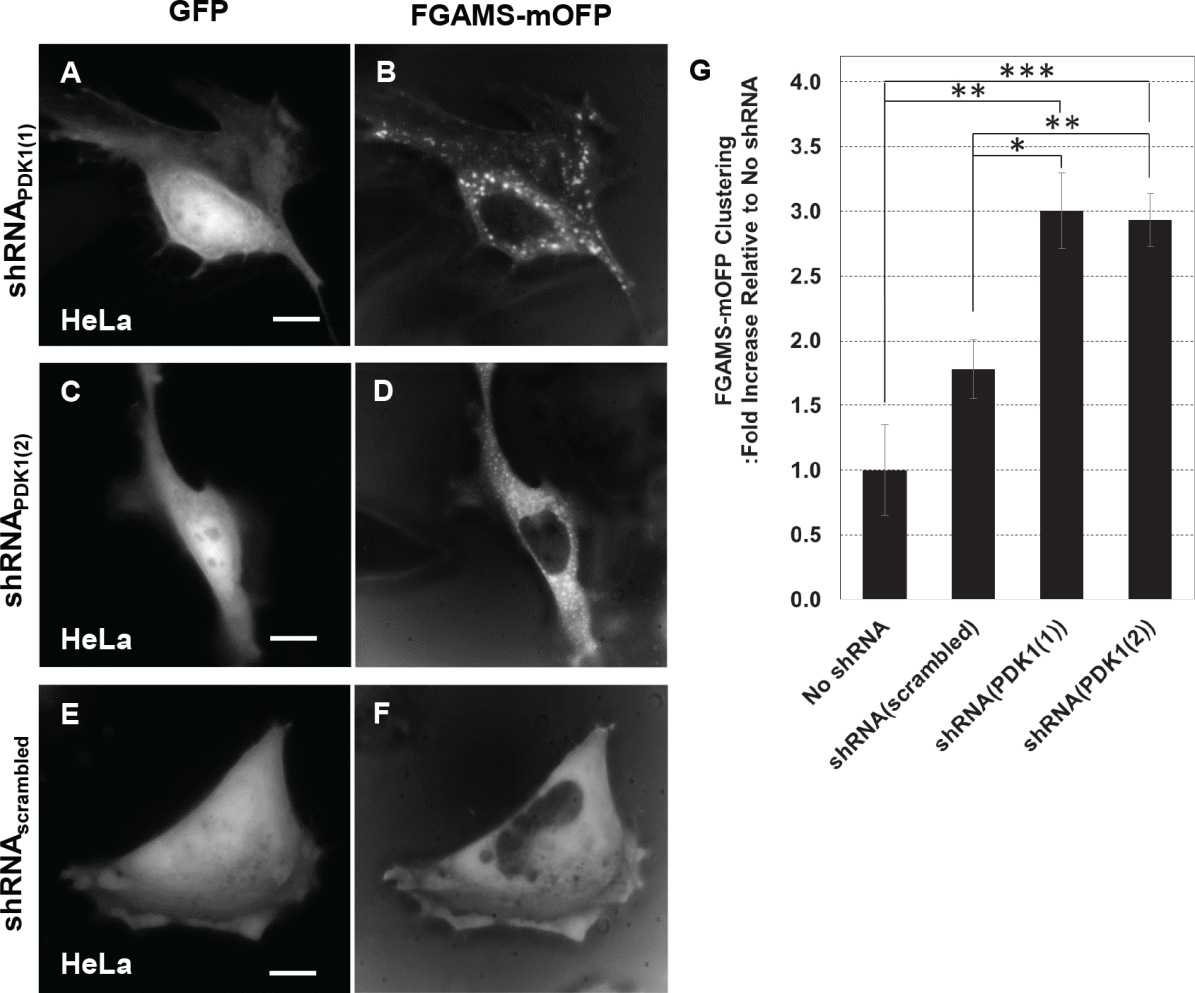
Knock-down of PDK1 induces the clustering of FGAMS-mEGFP. HeLa cells were co-transfected with shRNA_PDK1_ (A and C) and FGAMS-mOFP (B and D). As a control, HeLa cells were also co-transfected with scrambled shRNA in the presence of FGAMS-mOFP (E-F). The representative images were selected from at least five independent imaging sessions. The fold changes of FGAMS-mOFP clustering were quantified in the presence of shRNAs relative to no shRNA control (G). At least 270 cells were analyzed for each condition. Statistical analyses were performed using two-sample two-tail *t*-test. * *p* < 0.05, ** *p* < 0.01, *** *p* < 0.001. Scale bar, 10 μm.

To ensure that the observed result of FGAMS-mEGFP clustering in response to PDK1 inhibition was not due to off-target effects, we knocked down PDK1 using shRNA_PDK1_. We first performed western blot to reveal an overall knock-down efficiency of shRNA_PDK1_ in an ensemble level (S2 Fig). Then, HeLa cells were dually transfected with two different plasmids expressing FGAMS with monomeric orange fluorescent protein (FGAMS-mOFP) and shRNA_PDK1_. The bicistronic plasmid expressing shRNA_PDK1_ also encodes GFP as a transfection marker in single cells. Positive GFP signal from the transfected cell indicates the successful transfection of the plasmid expressing shRNA_PDK1_ in single-cell levels. As a result, we observed clustering of FGAMS-mOFP in the shRNA_PDK1_-expressing cells (Fig 3A–3D), but not in the shRNA_Scrambled_-transfected cells (Fig 3E–3F). Quantitatively, we observed a ~3-fold increase in FGAMS-mOFP clustering when co-expressed with shRNA_PDK1_ as compared to FGAMS-mOFP alone (Fig 3G). The formation of FGAMS-mOFP clusters also increased ~2-fold compared to the cells expressing scrambled shRNAs (Fig 3G). Collectively, these results support our conclusion that PDK1 plays a role in controlling cytoplasmic clustering of FGAMS in cells.

### Pharmacological inhibition of PI3K, Akt and mTOR had no impact on subcellular distribution of *de novo* purine biosynthetic enzymes

**Fig 4:**
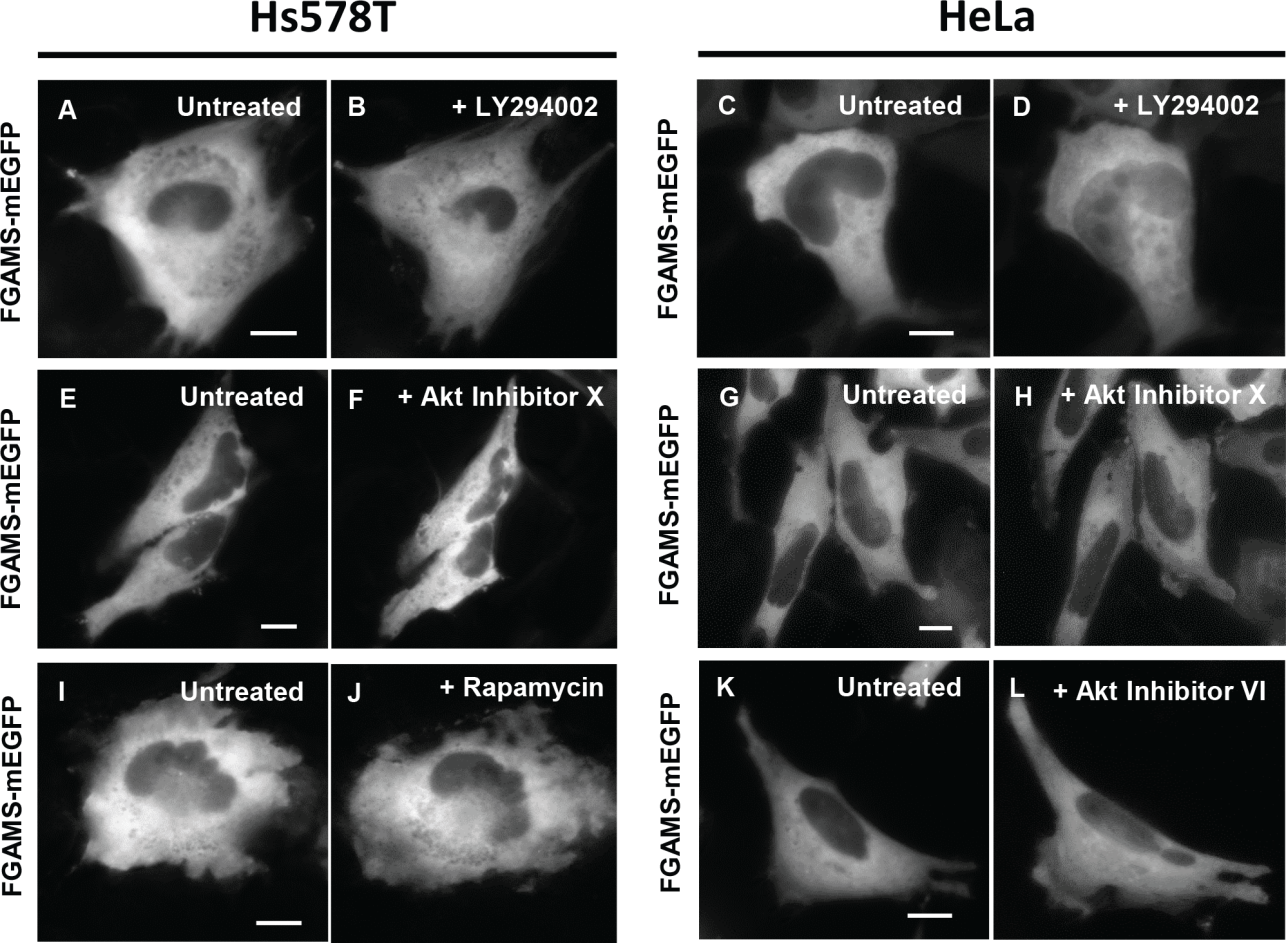
Inhibition of PI3K/Akt/mTOR signaling pathway has no effect on FGAMS-mEGFP localization. HeLa and Hs578T cells expressing FGAMS-mEGFP (A, C, E, G, I and K), were treated with inhibitors against PI3K (B and D, LY294002; *N*_HeLa_ = 200 and *N*_Hs578T_ = 408), Akt (F, H and L, Akt Inhibitor X; *N*_HeLa_ = 200 and *N*_Hs578T_ = 752 or VI; *N*_HeLa_ = 46), and mTOR (J, rapamycin; *N*_HeLa_ = 200). Treatment with each inhibitor did not change the subcellular localization of FGAMS-mEGFP. Cells were exposed to the small molecules for at least 4–5 hours. The representative images were selected from at least three independent imaging sessions. *N* indicates the number of the cells we have imaged in our study. Scale bar, 10 μm.

We have further treated cells expressing FGAMS-mEGFP with small molecules inhibiting protein kinases that are involved in the membrane-bound PDK1 signaling pathway. Briefly, LY294002 (26 μM) against PI3K, an inhibitor X (13 μM) or VI (0.5 μM) against Akt, and rapamycin (650 nM) against mTOR were individually administered to inhibit their target kinases in cells. However, no formation of FGAMS clusters in HeLa and Hs578T cells was observed in the presence of these inhibitors (Fig 4). Our negative effect of rapamycin on clustering of FGAMS-mEGFP in Hs578T cells grown in the nutrient-rich conditions is also consistent with the previous report that rapamycin treatment had no impact on the purinosome levels in HeLa cells [27]. In addition to FGAMS, we further tested the impact of Akt inhibitor X on the enzyme catalyzing other steps of *de novo* purine biosynthesis. However, TrifGART-GFP (steps 2, 3 and 5), ASL-mEGFP (step 8) and mEGFP-ATIC (steps 9 and 10) remained diffusive in the presence of Akt Inhibitor X in Hs578T cells, respectively (S3 Fig). Therefore, along with previous reports [23,27], our negative results against PI3K, Akt, and mTOR evidently rule out the possibility that the membrane-recruited PDK1 signaling cascades may regulate the spatial assemblies of purine biosynthetic enzymes.

### PDK1 inhibition induced the formation of the 3-enzyme core assembly but not the purinosome

FGAMS has been shown to participate in the formation of purinosomes for the upregulation of *de novo* purine biosynthesis [7] whereas AMP-activated protein kinase (AMPK) signaling networks downregulate *de novo* purine biosynthesis by promoting spatial sequestration of FGAMS into its own self-assembly in the cytoplasm [12]. We therefore have evaluated if the clustering of FGAMS-mEGFP is indicative of the purinosome [7] or the recently reported FGAMS self-assembly [12].

**Fig 5:**
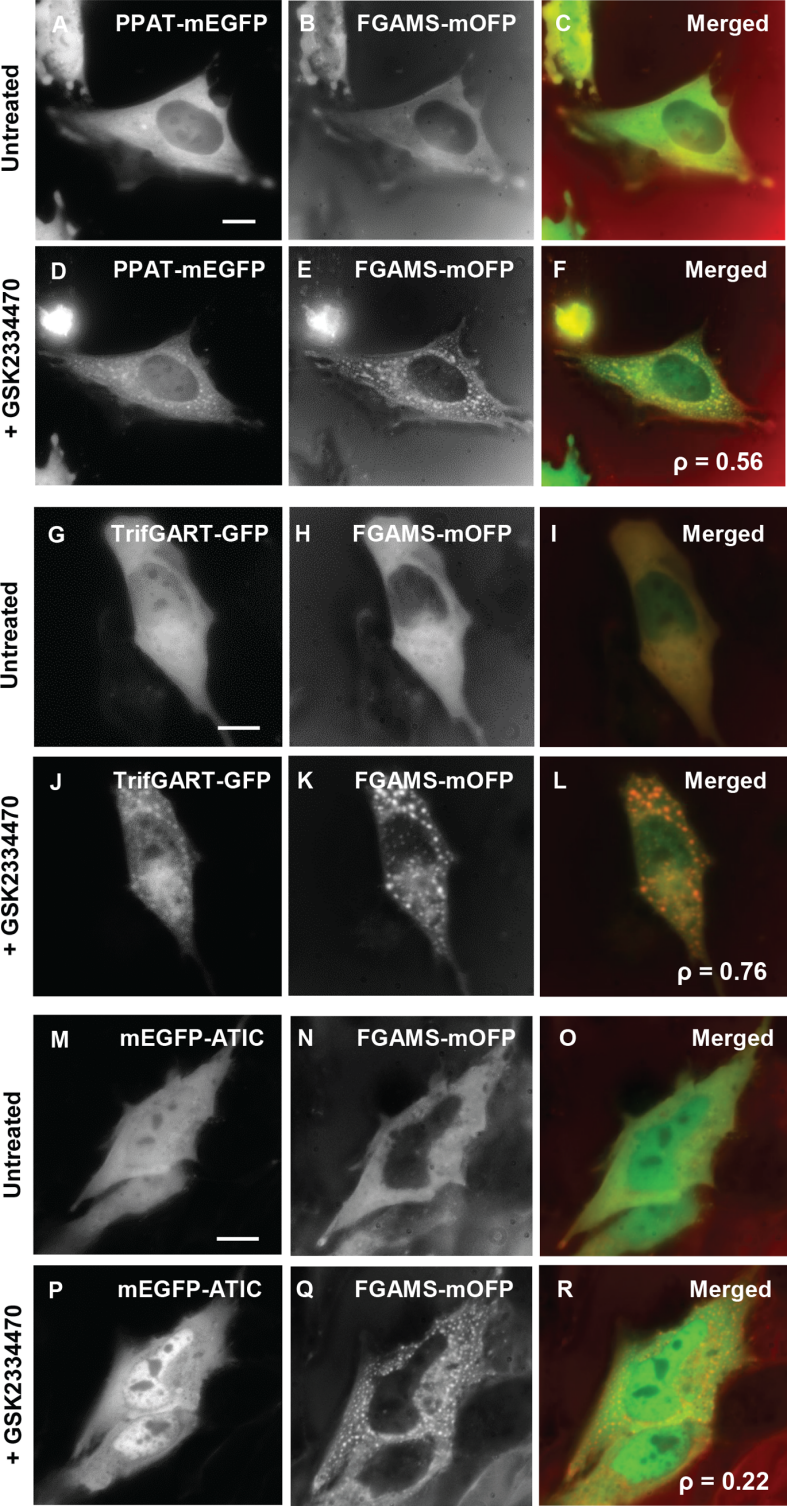
Pharmacological inhibition of PDK1 results in the formation of the 3-enzyme core assembly for *de novo* purine biosynthesis. HeLa cells expressing FGAMS-mOFP with PPAT-mEGFP (A-C) or TrifGART-GFP (G-I) promoted respective co-clusters in the presence of GSK2334470 (D-F and J-L, respectively) (*N*_Dually-Transfected-Cells_ > 80). As a control, HeLa cells expressing FGAMS-mOFP with mEGFP-ATIC (M-O), however, did not induce co-clusters in response to the treatment of GSK2334470 (P-R) (*N*_Dually-Transfected-Cells_ > 50). Cells were exposed to GSK2334470 for at least 4 hours. The representative images were selected from at least five independent imaging sessions. *N* indicates the number of the co-transfected cells we have imaged in our study. Pearson’s correlation coefficients (ρ) are indicated in merged images (F, L and R). Scale bar, 10 μm.

Since the purinosome consists of the 3-enzyme core assembly [8,9], the other GFP-tagged enzyme members of the core assembly, PPAT-mEGFP (step 1) and TrifGART-GFP (steps 2, 3 and 5), were co-transfected with FGAMS-mOFP. When we treated dually transfected cells with the PDK1 inhibitor, GSK2334470 (11 μM), colocalization of PPAT-mEGFP or TrifGART-GFP with FGAMS-mOFP was observed in 69 ± 15% or 51 ± 10% of co-transfected HeLa cells, respectively (Fig 5A–5L). It appears that 3 enzymes catalyzing the first half of the pathway (steps 1–5) form a spatial assembly in response to the PDK1 inhibition.

In addition, we examined colocalization of FGAMS-mOFP with mEGFP-ATIC (steps 9 and 10) to determine if the core assembly recruits the rest of *de novo* purine biosynthetic enzymes. However, after four hours with GSK2334470 (11 μM), mEGFP-ATIC remained diffusive while FGAMS-mOFP organized into clusters in HeLa cell (Fig 5M–5R). Similarly, ASL-mEGFP (step 8) did not assemble into cytoplasmic granules in the presence of GSK2334470 or Gö6983 in Hs578T cells, either (S4 Fig). Along with no influence of Akt inhibitor X on purine biosynthetic enzymes (Figs 4 and S3), we conclude that pharmacological inhibition of Akt-independent PDK1 activity induces the formation of the 3-enzyme assembly for *de novo* purine biosynthesis, which is clearly different from the purinosome and the FGAMS self-assembly.

Furthermore, we have performed fluorescence recovery after photobleaching (FRAP) experiments. Previously, we have used FRAP to characterize the internal structure of the purinosome, proposing the formation of a 3-enzyme core complex [8]. If the enzyme assembly we observed in the presence of GSK2334470 represents a 3-enzyme core assembly, we would anticipate FGAMS-mEGFP would have a similar apparent diffusion coefficient (D_*app*_) in GSK2334470-induced clusters as in the core assembly of the purinosome. Indeed, we found that the D_*app*_ of GSK2334470-induced FGAMS-mEGFP clusters is 0.005 ± 0.002 um^2^/sec (*N*_FRAP_ = 24) in Hs578T cells. This is similar to the previously reported D_*app*_ (0.007 ± 0.001 um^2^/sec) of the 3-enzyme core complex assembled inside the purinosome in Hs578T cells [8]. Therefore, it appears that the FGAMS-mEGFP clusters formed by GSK2334470 are similar to the core assemblies formed by FGAMS-mEGFP inside the purinosomes, collectively supporting our conclusion that the observed structure in the presence of GSK2334470 is a 3-enzyme core assembly.

### PDK1 inhibition also promoted clustering of endogenous enzymes in fixed HeLa cells

**Fig 6:**
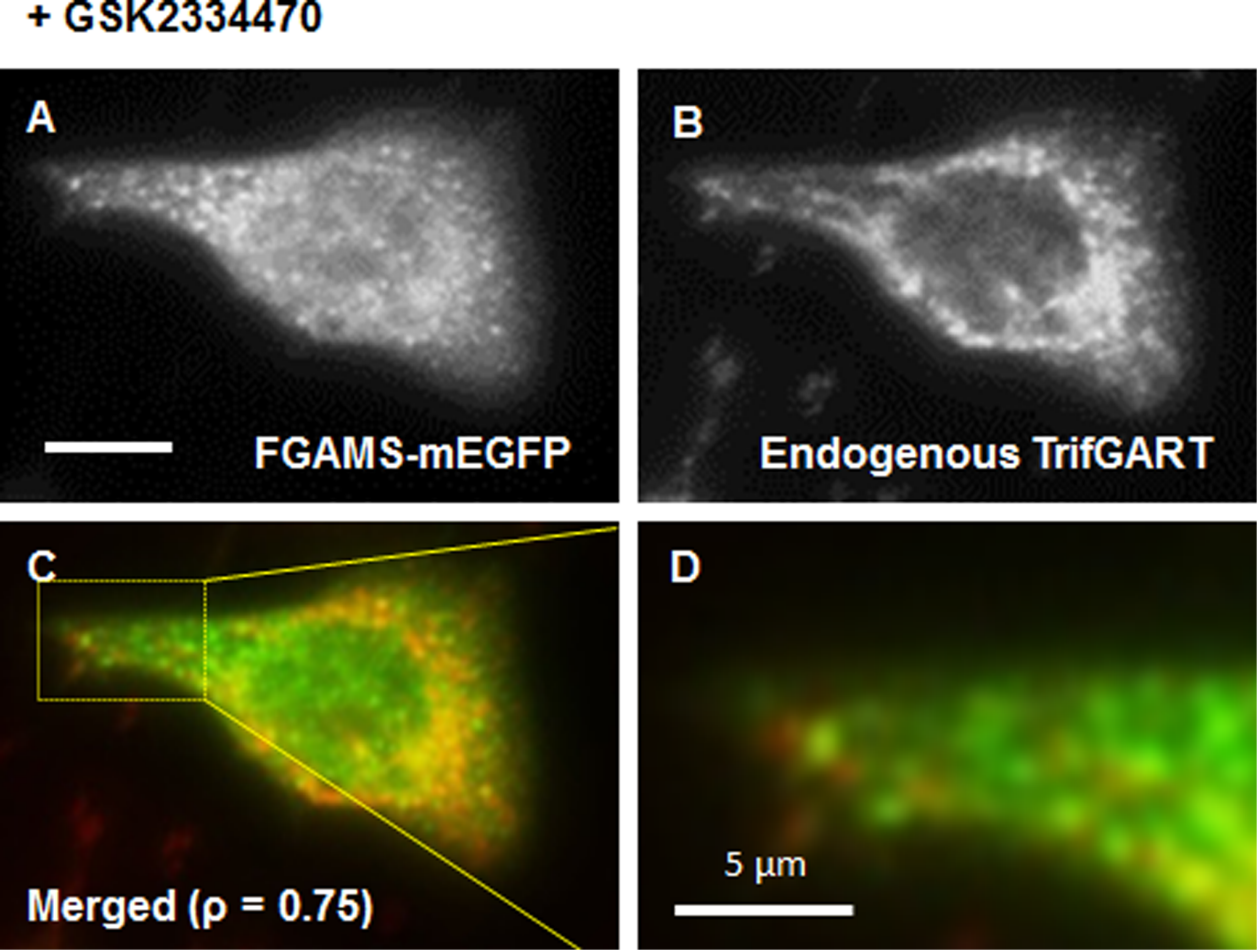
Immunocytochemistry of endogenous TrifGART with FGAMS-mEGFP in fixed HeLa cells. HeLa cells expressing FGAMS-mEGFP were treated with GSK2334470 (11–33 μM) and fixed with 100% methanol. The representative images demonstrate that clustered FGAMS-mEGFP (A) colocalizes with endogenous TrifGART (B) in fixed HeLa cells. Pearson’s correlation coefficient (ρ) is indicated in a merged image (C). A part of the cell is also zoomed in for clarification (D). At least 300 cells were analyzed. Scale bar, 10 μm, unless otherwise indicated.

We further investigated whether endogenous enzymes involved in *de novo* purine biosynthesis are spatially regulated in the presence of the PDK1 inhibitor. First, we imaged endogenous FGAMS in fixed HeLa cells before and after treatment of GSK2334470 (33 μM). Our immunofluorescence imaging clearly shows the promotion of FGAMS clusters in fixed HeLa cells (S5 Fig). Second, we also visualized colocalization of endogenous TrifGART with transfected FGAMS-mEGFP in fixed HeLa cells upon the treatment of 33 μM GSK2334470 (Fig 6). Along with our live-cell imaging, it is clear that PDK1 inhibition promotes the formation of the core assembly for the purinosome.

### The three-enzyme core assembly did not colocalize with the mitochondria or lipid membrane in Hs578T cells

To determine if the observed 3-enzyme core assembly is an independent cellular body from cellular organelles, we co-stained the core assembly with the mitochondria and lipid membrane in Hs578T cells. First, Hs578T cells expressing the core assembly after the treatment of GSK2334470 were subsequently stained with the mitochondria. Our imaging data clearly indicate no colocalization between the 3-enzyme core assembly and the mitochondria (Panel A-C S6 Fig). Second, we also stained the core assemblies in the presence of a lipid membrane dye (DiIC_16_(3)). However, we observed no apparent colocalization between the core assembly and lipid membrane-bound structures (Panel D-F in S6 Fig). Collectively, the observed 3-enzyme assemblies are spatially independent cellular granules from the mitochondira and other lipid-bound cellular structures.

## Discussion

In this work, we reveal the spatiotemporal alterations of *de novo* purine biosynthetic enzymes by Akt-independent PDK1 signaling pathways in human cancer cells. Pharmacological inhibition of PDK1 and its downstream substrates, PKC and S6K, promoted the formation of a 3-enzyme core assembly for *de novo* purine biosynthesis in HeLa and Hs578T cells. However, pharmacological inhibition of PI3K, Akt and mTOR, which are primarily associated with the plasma membrane-bound PDK1 activity, exhibited no impact on subcellular locations of *de novo* purine biosynthetic enzymes. Along with our shRNA-mediated knock-down study against PDK1, our data provide compelling evidence that the cytoplasmic activity of PDK1 was inhibited by GSK2334470, leading to the formation of the 3-enzyme core assembly. Hence, we demonstrate here that Akt-independent PDK1 signaling pathways regulate the subcellular localizations of *de novo* purine biosynthetic enzymes in cancer cells.

We are also aware that pharmacological inhibitors of protein kinases are notorious for off-target effects in cellular studies. Although the inhibition of GSK2334470 was proven to be highly specific to PDK1 (IC_50_, ~10 nM) after evaluating 94 protein kinases *in vitro* [14], higher doses of GSK2334470 showed its off-target effects on a few AGC protein kinases [14]. Hence, we have used both GSK2334470 and shRNA_PDK1_ to demonstrate the association of PDK1 with purine biosynthetic enzymes. In addition, our finding was further validated by targeting two downstream kinases of cytoplasmic PDK1 activity, PKC and S6K. Meanwhile, to support the proposed Akt-independent mechanism for the core assembly formation in this work, we have tested two different Akt inhibitors; a small-molecule inhibitor (Akt inhibitor X) and a peptide inhibitor (Akt inhibitor VI). Along with previously published data with another Akt inhibitor, A443654 (Abbott) [23], it is clear that Akt inhibition does not influence subcellular localization of *de novo* purine biosynthetic enzymes in HeLa and Hs578T cells. Collectively, this study strongly supports the molecular network between *de novo* purine biosynthetic enzymes and Akt-independent PDK1 signaling pathways in cancer cells.

However, the metabolic function of the 3-enzyme core assembly is not determined yet. Considering the promoted 3-enzyme core assembly contains the enzymatic activities of the first half (steps 1–5) of *de novo* purine biosynthesis, our data may suggest a potential function of the core assembly in controlling the direction of purine flux at the metabolic node between *de novo* purine biosynthesis and thiamine biosynthesis. This notion is not unprecedented by recent data showing that the spatial assemblies of metabolic enzymes in glucose metabolism are responsible for diverting glucose flux from glycolysis into anabolic biosynthetic pathways [28,29]. Along with our current knowledge of how the activity of *de novo* purine biosynthesis is regulated in single cells by the formation of the purinosome [10,11] or the FGAMS self-assembly [12], we propose that functional diversity of *de novo* purine biosynthesis may be accomplished by controlling the composition of metabolic enzymes into their spatial assemblies.

Lastly, Akt-independent PDK1 activity was recognized as a critical element in breast cancer metastasis [18,30]. Immunohistochemical analysis revealed the overexpression of PDK1 in more than 80% of breast cancer tissues samples examined [31]. A metastatic role of PDK1 in breast cancers was demonstrated to be independent of Akt activation in cell culture systems [18,30]. Importantly, pharmacological inhibition using GSK2334470 or shRNA-mediated knock-down of PDK1 decreased the viability, motility and malignancy of breast cancer cells in both cell culture and mouse models [19,20]. Therefore, the present study may suggest that the metabolic function of the 3-enzyme core assembly is controlled by the spatially resolved activity of PDK1 for cancer cell survival, migration and invasion.

## Supporting information

S1 Fig Non-specific effects of the PDK1 and PKC inhibitors are not detectable. HeLa and/or Hs578T cells expressing mEGFP alone were treated individually with GSK2334470 (*N*_HeLa_ = 158 and *N*_Hs578T_ = 12) or Gö6983 (*N*_HeLa_ = 85) (A-F). In addition, Hs578T cells expressing FGAMS-mEGFP were treated with DMSO, the vehicle control (G-H; *N*_Hs578T_ > 500). Cells were exposed to the small molecules for at least 4 hours. The representative images were selected from at least three independent imaging sessions. *N* indicates the number of the cells we have imaged in our study. Scale bar, 10 μm.
(TIF)

S2 Fig Western blot analysis of shRNA-mediated PDK1 knockdown. Lawns of HeLa cells were transfected with shRNA_PDK1 (1) or (2)_. After ~24 hours, cells were harvested for western blots for total PDK1 and actin. Negative controls include cell lysates that were transfected with shRNA_Scrambled_ or treated with no shRNA.
(TIF)

S3 Fig Inhibition of Akt does not affect the compartmentalization of purine biosynthetic enzymes in Hs578T cells. TrifGART-GFP (A-B; *N*_Hs578T_ = 39), ASL-mEGFP (C-D; *N*_Hs578T_ = 36) and mEGFP-ATIC (E-F; *N*_Hs578T_ = 164) showed no change in subcellular localization after treatment with Akt Inhibitor X. Cells were exposed to Akt inhibitor X for at least 4 hours. The representative images were selected from at least four independent imaging sessions. *N* indicates the number of the cells we have imaged in our study. Scale bar, 10 μm.
(TIF)

S4 Fig ASL-mEGFP does not cluster after treatment with PDK1 or PKC inhibitor in Hs578T cells. Treatment with GSK2334470 (A-B; *N*_Hs578T_ = 36) or Gö6983 (C-D; *N*_Hs578T_ = 120) for 4–5 hours did not result in the spatial alteration of ASL-mEGFP in cells. The representative images were selected from at least three independent imaging sessions. *N* indicates the number of the cells we have imaged in our study. Scale bar, 10 μm.
(TIF)

S5 Fig Endogenous FGAMS forms cytoplasmic clusters following PDK1 inhibition. HeLa cells were treated with GSK2334470 for 4 hours, and fixed and immunostained for endogenous FGAMS. The representative images were selected from at least three independent imaging sessions. At least 300 cells were analyzed. Scale bar, 10 μm.
(TIF)

S6 Fig Localization of the 3-enzyme core assembly with other cellular compartments. Hs578T cells expressing FGAMS-mEGFP were treated with GSK2334470 (A and D) for 4 hours to promote the core assembly, and subsequently stained for the mitochondria (B) with MitoTracker Orange or lipids (E) using DiIC_16_. The representative images were selected from at least three independent imaging sessions. At least 100 cells were analyzed. Scale bar, 10 μm.
(TIF)
