# Supplementary figures and images for: Spatial alterations of De Novo purine biosynthetic enzymes by Akt-independent PDK1 signaling pathways
Source: PLoS One. 2018 Apr 18;13(4):e0195989. doi: 10.1371/journal.pone.0195989 (PMC5905998; doi:10.1371/journal.pone.0195989)

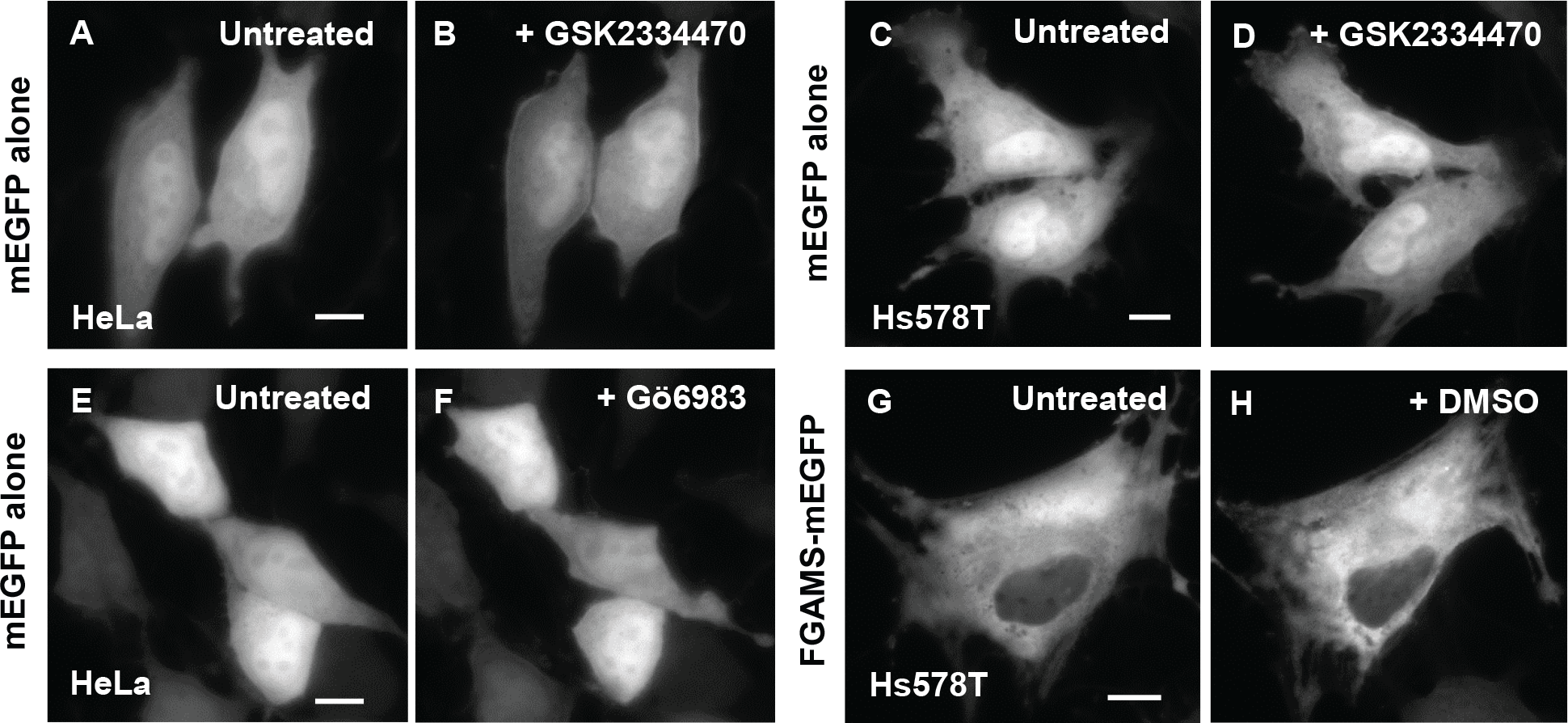

Supplement: S1 Fig — HeLa and/or Hs578T cells expressing mEGFP alone were treated individually with GSK2334470 (NHeLa = 158 and NHs578T = 12) or Gö6983 (NHeLa = 85) (A-F). In addition, Hs578T cells expressing FGAMS-mEGFP were treated with DMSO, the vehicle control (G-H; NHs578T > 500). Cells were exposed to the small molecules for at least 4 hours. The representative images were selected from at least three independent imaging sessions. N indicates the number of the cells we have imaged in our study. Scale bar, 10 μm. (TIF) [file pone.0195989.s001.tif]

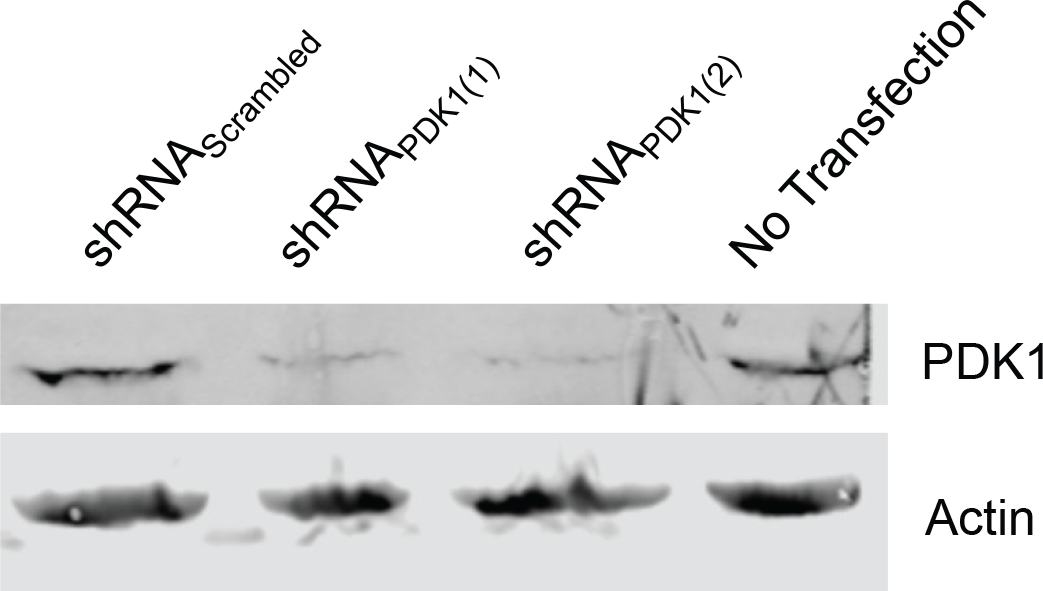

Supplement: S2 Fig — Lawns of HeLa cells were transfected with shRNAPDK1 (1) or (2). After ~24 hours, cells were harvested for western blots for total PDK1 and actin. Negative controls include cell lysates that were transfected with shRNAScrambled or treated with no shRNA. (TIF) [file pone.0195989.s002.tif]

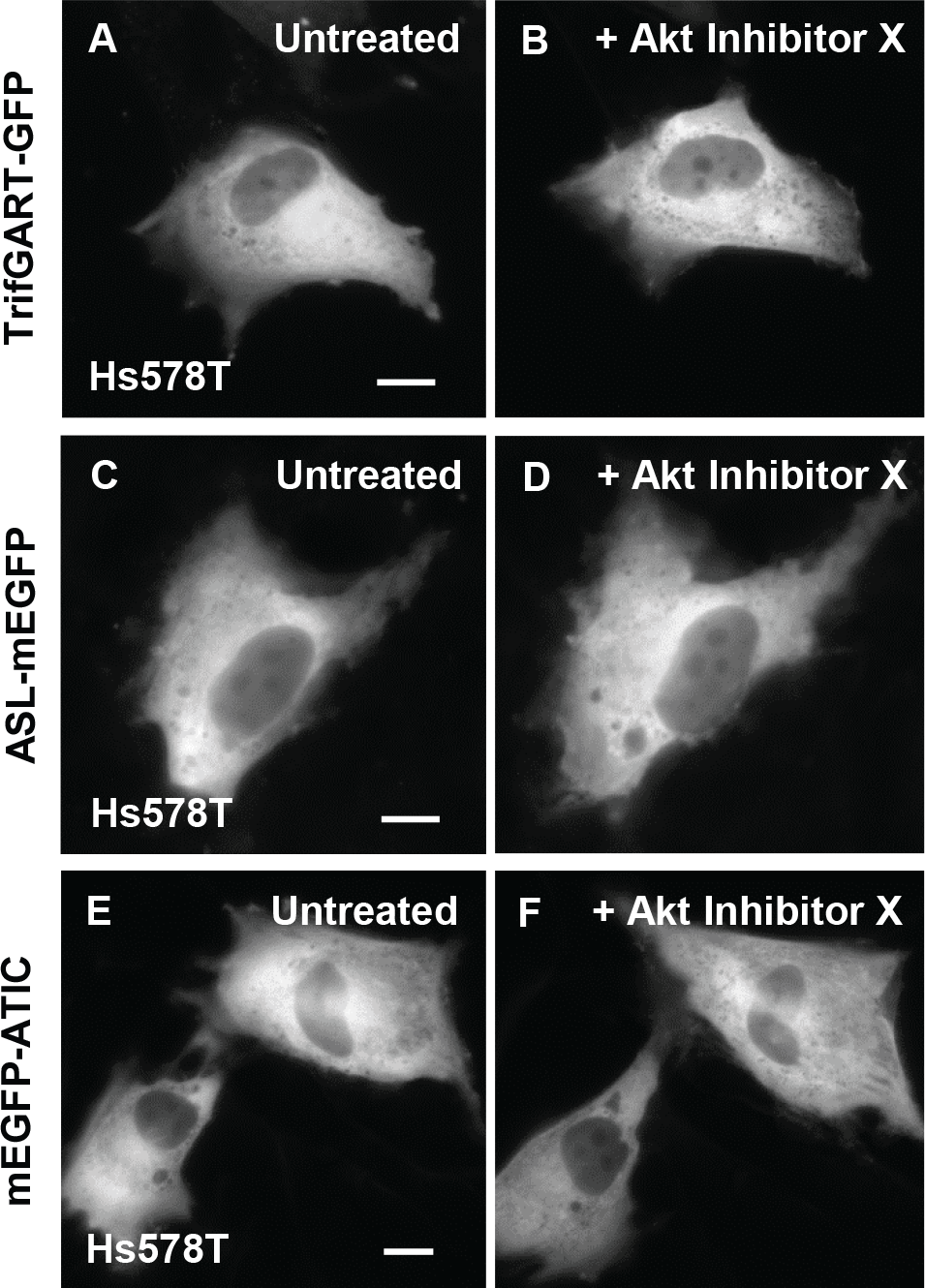

Supplement: S3 Fig — TrifGART-GFP (A-B; NHs578T = 39), ASL-mEGFP (C-D; NHs578T = 36) and mEGFP-ATIC (E-F; NHs578T = 164) showed no change in subcellular localization after treatment with Akt Inhibitor X. Cells were exposed to Akt inhibitor X for at least 4 hours. The representative images were selected from at least four independent imaging sessions. N indicates the number of the cells we have imaged in our study. Scale bar, 10 μm. (TIF) [file pone.0195989.s003.tif]

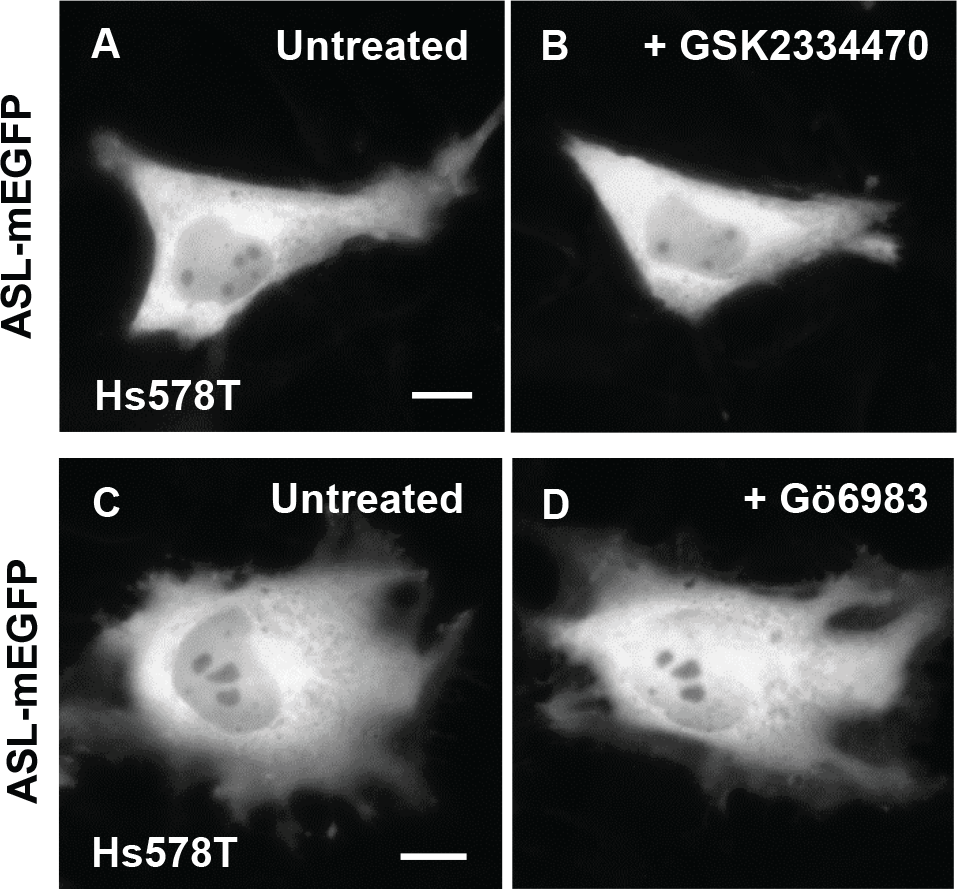

Supplement: S4 Fig — Treatment with GSK2334470 (A-B; NHs578T = 36) or Gö6983 (C-D; NHs578T = 120) for 4–5 hours did not result in the spatial alteration of ASL-mEGFP in cells. The representative images were selected from at least three independent imaging sessions. N indicates the number of the cells we have imaged in our study. Scale bar, 10 μm. (TIF) [file pone.0195989.s004.tif]

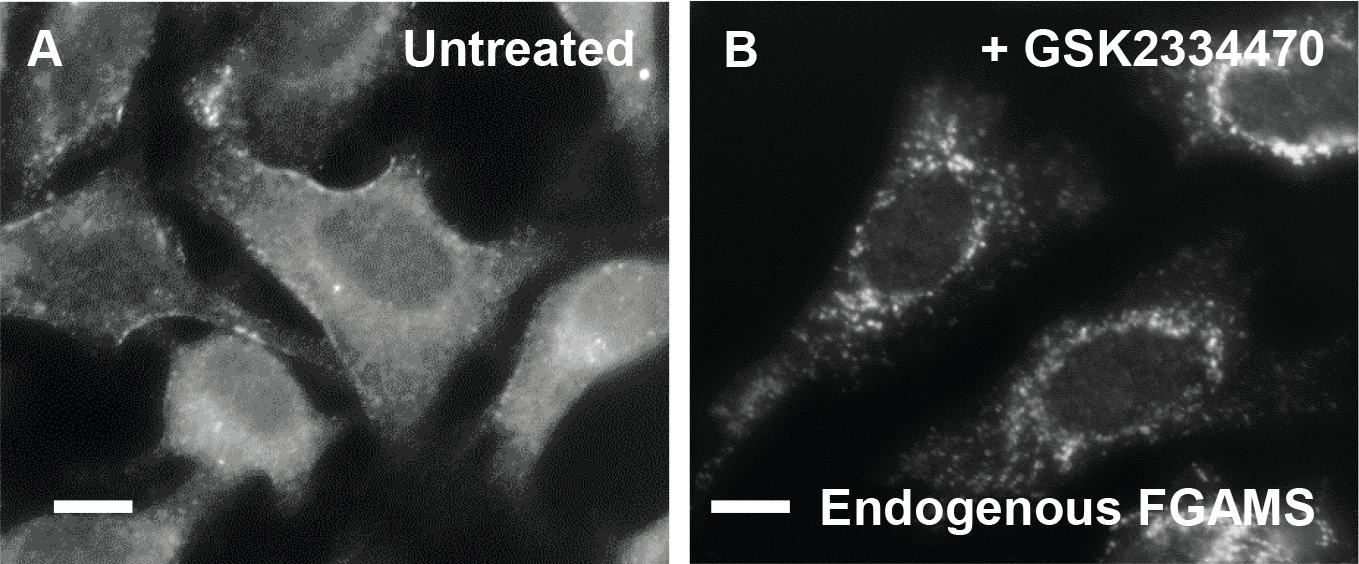

Supplement: S5 Fig — HeLa cells were treated with GSK2334470 for 4 hours, and fixed and immunostained for endogenous FGAMS. The representative images were selected from at least three independent imaging sessions. At least 300 cells were analyzed. Scale bar, 10 μm. (TIF) [file pone.0195989.s005.tif]

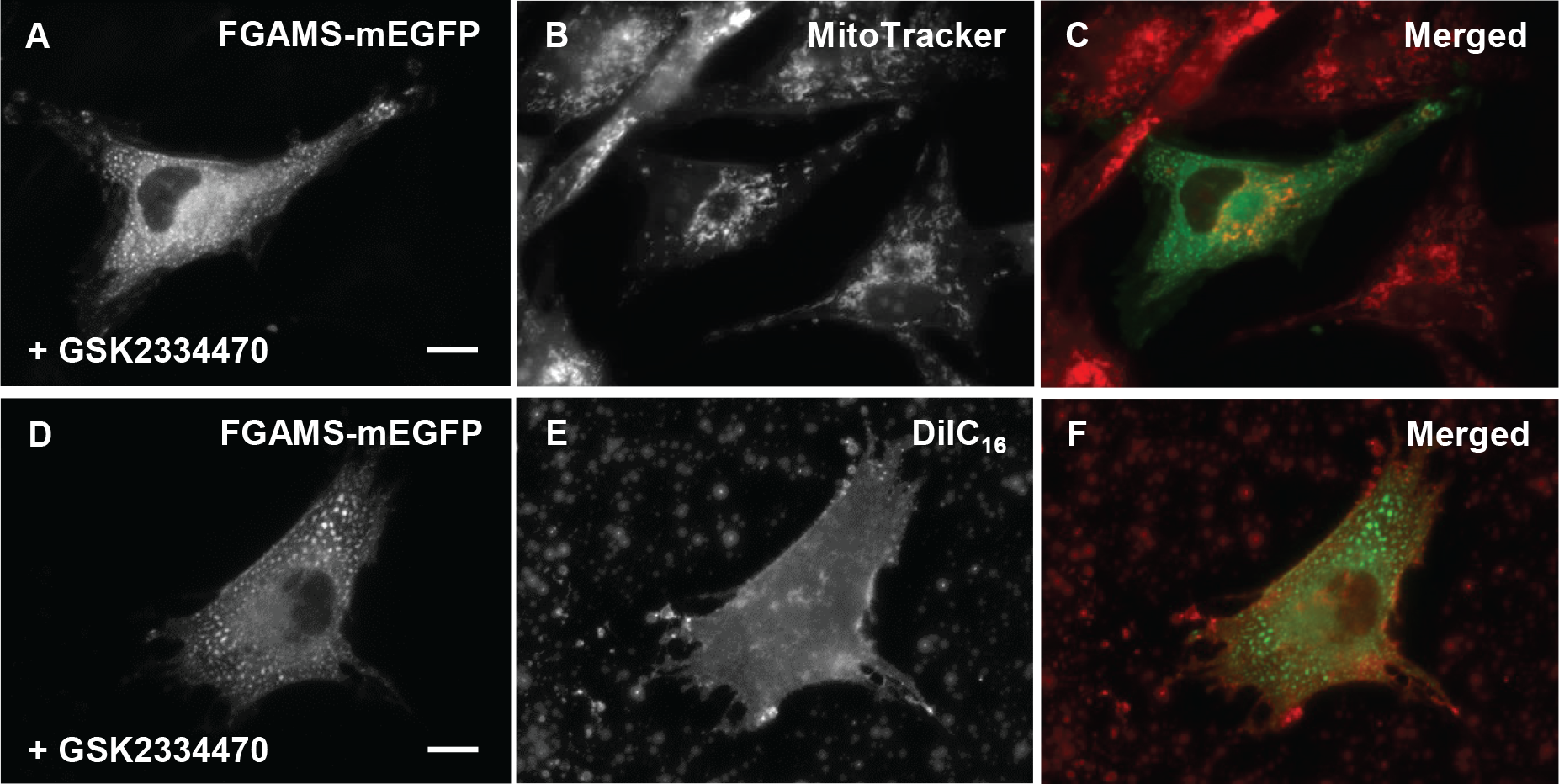

Supplement: S6 Fig — Hs578T cells expressing FGAMS-mEGFP were treated with GSK2334470 (A and D) for 4 hours to promote the core assembly, and subsequently stained for the mitochondria (B) with MitoTracker Orange or lipids (E) using DiIC16. The representative images were selected from at least three independent imaging sessions. At least 100 cells were analyzed. Scale bar, 10 μm. (TIF) [file pone.0195989.s006.tif]
